# ROS scavengers decrease γH2ax spots in motor neuronal nuclei of ALS model mice *in vitro*

**DOI:** 10.3389/fncel.2022.963169

**Published:** 2022-08-31

**Authors:** Maya Junghans, Felix John, Hilal Cihankaya, Daniel Schliebs, Konstanze F. Winklhofer, Verian Bader, Johann Matschke, Carsten Theiss, Veronika Matschke

**Affiliations:** ^1^Department of Cytology, Institute of Anatomy, Ruhr University Bochum, Bochum, Germany; ^2^Department of Molecular Cell Biology, Institute of Biochemistry and Pathobiochemistry, Ruhr University Bochum, Bochum, Germany; ^3^Cluster of Excellence RESOLV, Bochum, Germany; ^4^Department of Biochemistry of Neurodegenerative Diseases, Institute of Biochemistry and Pathobiochemistry, Ruhr University Bochum, Bochum, Germany; ^5^Institute of Cell Biology (Cancer Research), University Hospital Essen, University of Duisburg-Essen, Essen, Germany

**Keywords:** Wobbler, double strand breaks, glutathione ethyl ester, N-Acetyl-L-Cysteine, Mito-TEMPO, p53bp1, neuroprotection

## Abstract

**Background:** Amyotrophic lateral sclerosis (ALS) is an incurable neurodegenerative disease characterized by the loss of motor neurons in cerebral cortex, brainstem and spinal cord. Numerous studies have demonstrated signs of oxidative stress in postmortem neuronal tissue, cerebrospinal fluid, plasma and urine of ALS patients, without focusing on the specific processes within motor neurons. Thus, we aimed to investigate the relevance of reactive oxygen species (ROS) detoxification mechanisms and its consequences on the formation of toxic/lethal DNA double strand breaks (DSBs) in the ALS model of the Wobbler mouse.

**Methods:** Live cell imaging in dissociated motor neuronal cultures was used to investigate the production of ROS using Dihydroethidium (DHE). The expression levels of ROS detoxifying molecules were investigated by qPCR as well as Western blots. Furthermore, the expression levels of DNA damage response proteins p53bp1 and H2ax were investigated using qPCR and immunofluorescence staining. Proof-of-principle experiments using ROS scavengers were performed *in vitro* to decipher the influence of ROS on the formation of DNA double strand breaks quantifying the γH2ax spots formation.

**Results:** Here, we verified an elevated ROS-level in spinal motor neurons of symptomatic Wobbler mice *in vitro*. As a result, an increased number of DNA damage response proteins p53bp1 and γH2ax in dissociated motor neurons of the spinal cord of Wobbler mice was observed. Furthermore, we found a significantly altered expression of several antioxidant molecules in the spinal cord of Wobbler mice, suggesting a deficit in ROS detoxification mechanisms. This hypothesis could be verified by using ROS scavenger molecules *in vitro* to reduce the number of γH2ax foci in dissociated motor neurons and thus counteract the harmful effects of ROS.

**Conclusion:** Our data indicate that maintenance of redox homeostasis may play a key role in the therapy of the neurodegenerative disease ALS. Our results underline a necessity for multimodal treatment approaches to prolong the average lifespan of motor neurons and thus slow down the progression of the disease, since a focused intervention in one pathomechanism seems to be insufficient in ALS therapy.

## Introduction

Amyotrophic lateral sclerosis (ALS) is the most common motor neuron disease in adults with a prevalence of 3–5/100,000 in Europe and the United States (Brown and Al-Chalabi, 2017). About 5%–10% of ALS patients are diagnosed with the hereditary, familial form (fALS) and 90%–95% with the sporadic form of ALS (sALS). In individual cases a clear classification to a specific subtype is often difficult (Al-Chalabi et al., 2016). This neurodegenerative disease is characterized by the loss of both, the upper motor neurons in the cerebral cortex and the lower motor neurons in the brainstem and spinal cord (Brown and Al-Chalabi, 2017; Petrov et al., 2017).

ALS is rapidly progressive and 50% of all ALS patients die in the first 30 months after symptoms appear, often due to respiratory failure (Kiernan et al., 2011). Although ALS was first described by Charcot and Joffroy in 1869, the causality of the disease is still poorly understood (Petrov et al., 2017). In recent decades, ALS research has identified a great heterogeneity in pathological abnormalities between different ALS subgroups like modified protein homeostasis, excitotoxicity, modified RNA homeostasis, neuroinflammation, dysfunctional axonal transport, mitochondrial dysfunction, and oxidative stress (Brown and Al-Chalabi, 2017; Tam et al., 2019). Currently there are only two drugs approved for the treatment of ALS: Riluzole, an anti-glutamatergic agent, extending the life of ALS patients for approximately 3 months (Jaiswal, 2019), and Edaravone, an antioxidant and free-radical scavenger (Brown and Al-Chalabi, 2017; Jaiswal, 2019), with its capability to inhibit ALS motor deterioration and thus slowing disease progression in a subpopulation of ALS patients, especially patients with shorter duration and milder symptoms (Abe et al., 2014; Yoshino, 2019).

Various mouse models with different ALS-associated mutations are currently available to study the pathomechanisms of ALS (Stephenson and Amor, 2017; Cihankaya et al., 2021), including the Wobbler mouse, which was first described by Falconer in 1956 (Andrews et al., 1974; Giorgio et al., 2019). The Wobbler mouse displays almost the same symptoms as human ALS patients at the clinical-symptomatic, cellular and metabolic levels (Boillée et al., 2003; Moser et al., 2013; Ott et al., 2015). A spontaneous autosomal recessive missense mutation L967Q located in the Vps54 gene, mapped to mouse chromosome 11, is responsible for the Wobbler phenotype (Kaupmann et al., 1992; Schmitt-John et al., 2005). The development of the clinical phenotype of Wobbler mice can be divided into three different phases (Ott et al., 2015). During the first pre-symptomatic phase, from p0 to p19, Wobbler mice cannot be distinguished from their wild-type and heterozygous siblings (Boillée et al., 2003). This is followed by the evolutionary phase from p20 to p39 which is characterized by a development of symptoms. After forty days the disease development proceeds with the stabilized clinical phase which is characterized by a stagnation of symptoms (Duchen and Strich, 1968; Ott et al., 2015; Saberi et al., 2016). The fully developed phenotype of the homozygous Wobbler mouse shows a head tremor, a wobbly gait pattern, an inability to climb and an overall muscle atrophy as well as a reduced body size and weight compared to wild-type mice (Ott et al., 2015).

Recent studies described the importance of oxidative stress for the pathogenesis, both in Wobbler mice and in ALS patients (Petrov et al., 2017; Röderer et al., 2018; Matschke et al., 2019; Tam et al., 2019; Wang et al., 2019; Zwilling et al., 2020; Stein et al., 2021). Our previous studies identified significantly increased levels of reactive oxygen species (ROS) as a marker for oxidative stress in the stable clinical phase (p40) of the Wobbler spinal cord (Röderer et al., 2018). In many cases, ROS arise as physiological molecules (Gorrini et al., 2013; Pourahmad et al., 2016; Matschke et al., 2019). Depending on their concentration, ROS influence various biological processes, and a balanced intracellular ROS concentration is essential for a continuously functioning cell metabolism (Rhee, 2006; Janssen-Heininger et al., 2008; Brieger et al., 2012; Matschke et al., 2019). However, excessive ROS production or a failure in defense mechanisms is extremely harmful to the cell as components such as DNA, RNA, proteins and lipids are oxidized. ROS can lead to single-strand (SSB) or double strand breaks (DSB) of the DNA *via* various factors. Among others, ROS can directly lead to DNA SSB through the oxidation of bases. In case of strongly increased ROS concentrations, these types of damage can occur in close proximity to each other on both DNA strands, resulting in DNA DSB (Cannan et al., 2014; Henrikus et al., 2019; Srinivas et al., 2019). Mitotic cells can counter such damage effectively and accurately by homologous recombination. However, post-mitotic cells, such as neurons, may respond to such damage only through rather error-prone but essential mechanisms such as non-homologous end joining (NHEJ; Lieber, 2010; Iyama and Wilson, 2013; Shanbhag et al., 2019). Oxidative DNA damage in post-mitotic cells might therefore be particularly dangerous for the survival of neuronal cells, as they can accumulate with age and lead to cell death (Islam, 2017). To counteract oxidative stress and thus oxidative DNA damage under physiological conditions the cell is equipped with various antioxidant mechanisms to scavenge ROS and maintain redox homeostasis, which is essential for normal cell function (Lagouge and Larsson, 2013; Pourahmad et al., 2016; Matschke et al., 2019).

Recent studies have already tried to overcome oxidative stress in Wobbler mice. Edaravone, as a free radical scavenger delays the disease progression in Wobbler mice and is already an approved drug for ALS patients in Japan, South Korea, Canada and the US (Ikeda and Iwasaki, 2015; Yoshino, 2019). N-Acetyl-L-Cysteine (NAC) can also act as an antioxidant—either directly or as a precursor for the antioxidant glutathione (Henderson et al., 1996). The degeneration of Wobbler mice motor neurons, shown by axon caliber, muscle size, muscle area and forelimb functionality, could significantly be decreased by giving the mice NAC-supplemented water (Henderson et al., 1996). In addition, Wobbler mice exposed to intraperitoneally high doses of the antioxidant Methylcarbylamine experienced a reduced rate of symptom progression compared to non-treated mice (Ikeda et al., 2015).

Since several studies observed a delay in symptomatic and pathological progression of motor neuron disease in Wobbler mice by the use of antioxidant molecules, we hypothesize that a dysfunction of antioxidant mechanisms may be the reason for increased ROS levels in spinal cord of this mouse model. Therefore, we aimed to investigate the underlying processes and mechanisms for the increased oxidative stress in motor neurons of Wobbler mice. Here, we deciphered the role of the redox homeostasis in Wobbler spinal cord and dissociated motor neuronal enriched cell culture on the formation of toxic DNA double strand breaks (DNBs) as well as the impact of ROS scavengers on the prevention of integrity of Wobbler motor neuron DNA.

## Materials and Methods

### Animals

All procedures were conducted under established standards of the German federal state of North Rhine Westphalia, in accordance with the European Communities Council Directive 2010/63/EU on the protection of animals used for scientific purposes. Animal experiments were carried out according to the German animal welfare regulations and approved by the local authorities (registration number Az. 84-02.04.2017.A085). As described earlier, for all experiments the mouse strain C57BL/Fa carrying the Wobbler mutation was used (Ott et al., 2015). Breeding, handling and genotyping of mice was performed as previously described (Ott et al., 2015). For each experiment, the spinal cord of both homozygous genotypes (wild-type and Wobbler) and gender was collected. Since our focus was to investigate oxidative DNA damage, only tissue from the stable clinical stage (p40) was used, as we previously showed no oxidative stress in the Wobbler spinal cord at earlier time points (Röderer et al., 2018). Heterozygous animals were only used for breeding.

### Immunofluorescence staining of DNA damage response proteins

To investigate whether the cervical spinal cord of Wobbler mice exhibits increased expression of DNA damage response proteins p53bp1 and phosphorylated H2ax (γH2ax), immunofluorescence staining on sections of five animals per genotype was performed. For this purpose animals (p40) were anesthetized with ketamine (100 mg/kg) and xylazine (10 mg/kg) and perfused with 4% PFA in PBS, as described before (Klatt et al., 2019). After decapitation and dissection of the cervical spinal cord, the tissue was post fixated with 4% PFA in PBS at 4°C for 24 h, transferred to 30% sucrose for 48 h and frozen in isopentane at −45°C (Klatt et al., 2019). Afterwards the tissue was embedded in tissue freezing medium (#14020108926, Leica Biosystems Inc., Buffalo Grove, Illinois, USA). Ten μm thick slices of cervical spinal cord were cut on a cryostat (Thermo Fisher Scientific, CryoStar NX50 Cryostat, chamber temperature −18°C; stage temperature −20°C). Cryosections were applied on SuperFrost^®^ Plus objective slides (Thermo Fisher Scientific, Waltham, Massachusetts, USA) and dried at 37°C for 30 min.

For immunofluorescence staining, cryosections were incubated for 1 min with PBS followed by 60 min incubation with 0.3% Triton-X100 and 5% goat serum in PBS. Further, the sections were incubated with the primary antibody p53bp1 (1:100 in 5% goat serum in PBS, #sc-22760, Santa-Cruz Biotechnology, Dallas, Texas, USA) or γH2ax (1:200 in 5% goat serum in PBS, #2577, Cell Signaling Technology, Danvers, Massachusetts, USA) at 4°C over night. After washing with PBS, the sections were reacted with the secondary antibody anti-rabbit-AlexaFluor488 (1:200 in PBS, #A11008, Thermo Fisher Scientific, Waltham, Massachusetts, USA) for 2 h at room temperature. Nuclei were stained with DAPI (1μg/ml; #D9542, Sigma-Aldrich, Danvers, Massachusetts, USA) for 30 min. Finally, the slices were covered with fluoroshield (#F6937, Sigma-Aldrich, St. Louis, Missouri, USA) and stored at 4°C.

Samples were imaged using an inverted confocal Laser Scanning Microscope (LSM 800, Carl Zeiss Microscopy GmbH, Jena, Germany) equipped with the respective filter sets in combination with a 10× (Plan-Apochromat 10×/0.45, Carl Zeiss Microscopy GmbH, Jena, Germany) and 40× objective (Plan-Apochromat 40×/1.4 Oil, Carl Zeiss Microscopy GmbH, Jena, Germany). Fluorescence image acquisition settings were kept the same for all conditions to achieve quantitative comparability of p53bp1 or γH2ax signals. Secondary antibodies were tested for specificity and showed no unspecific binding. ImageJ 1.53f51 (National Institute of Health, USA) was used to analyze the fluorescence intensity of p53bp1 and γH2ax in cervical spinal cord sections. Fluorescence signals of p53bp1 or γH2ax were measured in the nuclear area of individual cells, normalized to the respective DNA content (integrated DAPI signals) and expressed as relative fluorescence units (RFU) representing the relative levels of p53bp1 or γH2ax.

### RNA isolation, reverse transcription, and quantitative real-time PCR

RNA isolation, Reverse Transcription (RT) and qPCR were performed as described earlier (Rohm et al., 2019). Total RNA was extracted from the cervical spinal cord tissue of five Wobbler and five wild-type mice at stable clinical phase (p40) using the NucleoSpin^®^ miRNA isolation kit (# 740304, Macherey-Nagel, Düren, Germany) according to the manufacturer’s protocol for animal tissue and cultured cells. cDNA was synthesized using qScript cDNA SuperMix (#95048, QuantaBio, Beverly, Massachusetts, USA) according to the manufacturer’s protocol using 500 ng total RNA and oligo(dT) primer. Quantitative real-time PCR was performed on a CFX96 real-time PCR Detection System (Bio-Rad, Hercules, California, USA) using GoTaq qPCR Master Mix (#A6001, Promega, Madison, Wisconsin, USA). Specific primer used are shown in Table 1. Expression levels were analyzed in three independent qPCR runs in triplicates and normalized to *GAPDH*. The obtained Ct-values were analyzed using the 2^−ΔΔCt^ method (Livak and Schmittgen, 2001).

**Table 1:** Primer sequences used for quantitative real-time PCR.

**Gene**	**Forward**	**Reversed**
Glyceraldehyde 3-phosphate dehydrogenase (*GAPDH)*	5’-GGA GAA ACC TGC CAA GTA TGA-3’	5’-TCC TCA GTG TAG CCC AAG A-3’
Tumor protein p53 binding protein one *(Tp53bp1)*	5’-GAT AGA ACA GCC CAG CAA AGA-3’	5’-TCG GAC CTT ACA AGT GGT AGA-3’
Histone 2ax (*H2ax)*	5’-ATG TGA ACC CAG TTT CTC TAG G-3’	5’-CGG CAG GTA TAG AAC TCT TGT C-3’
Superoxide dismutase 1 *(SOD1)*	5’-CGG TGA ACC AGT TGT GTT GTC-3’	5’-CTG CAC TGG TAC AGC CTT GT-3’
Superoxide dismutase 2 *(SOD2)*	5’-TTC TGG ACA AAC CTG AGC CC-3’	5’-CGG CTG TCA GCT TCT CCT TA-3’
Catalase *(Cat)*	5’-CCT CGT TCA GGA TGT GGT TT-3’	5’-CGT GGG TGA CCT CAA AGT ATC-3’
Glutathione peroxidase 4 *(GPX4)*	5’-GCC CGA TAT GCT GAG TGT GG-3’	5’-CGG CTG CAA ACT CCT TGA TTT-3’

### SDS-gel electrophoresis and Western blotting

SDS-gel electrophoresis and Western blotting were performed as previously described (Stein et al., 2021). For SDS-gel electrophoresis proteins were isolated from four to eight cervical spinal cord samples (p40) per genotype using Cell lysis buffer (#9803S, Cell Signaling Technology, Danvers, Massachusetts, USA) supplemented with protease inhibitor (#11697498001, Merck, Darmstadt, Germany). Protein concentrations were determined by Pierce^TM^ BCA Protein Assay Kit (#23225, Thermo Fisher Scientific, Waltham, Massachusetts, USA). Fifty microgram of total protein was separated by SDS-gel electrophoresis and transferred to a nitrocellulose membrane. Blots were blocked with 1% RotiBlock (#A151, Roth, Karlsruhe, Germany) in 1× TBS for 1 h at room temperature. Primary antibodies (Table 2) were incubated over night at 4°C. HRP-coupled secondary antibodies (Table 2) were incubated for 1 h at room temperature. Finally, Immuno Cruz Luminol Agent (#sc-2048, Santa Cruz Biotechnology, Dallas, Texas, USA) was used for signal detection with an imaging system (ChemiDoc XRS+, Bio-Rad, Hercules, California, USA). For arithmetic analysis of the band intensity ImageJ 1.53f51 (National Institute of Health, USA) software was used. Band intensities of proteins of interest were normalized to the housekeeper actin. Normalized protein levels were compared between different genotypes.

**Table 2:** Primary and secondary antibodies used for Western blotting.

**Antibody**	**Dilution**	**Order number**
Anti-actin, rabbit polyclonal IgG antibody	1:250 in Roti-TBS	#A5060, Sigma-Aldrich, St. Louis, Missouri, USA
Anti-SOD1, mouse monoclonal IgG antibody	1:200 in Roti-TBS	#sc-101523, Santa Cruz Bio-technology, Dallas, Texas, USA
Anti-SOD2, mouse monoclonal IgG antibody	1:100 in Roti-TBS	#sc-133134, Santa Cruz Bio-technology, Dallas, Texas, USA
Anti-GPX4, mouse monoclonal IgG antibody	1:200 in Roti-TBS	#sc-166570, Santa Cruz Bio-Dallas, Texas, USA
Anti-Catalase, mouse monoclonal IgG antibody	1:200 in Roti-TBS	#sc-271803, Santa Cruz Bio-technology, Dallas, Texas, USA
Goat anti-mouse IgG (H+L)-HRP conjugate	1:10,000 in Roti-TBS	#1706516, Bio-Rad, Hercules, California, USA
Goat anti-rabbit IgG (H+L)-HRP conjugate	1:10,000 in Roti-TBS	#1706515, Bio-Rad, Hercules, California, USA

### Motor neuron enriched dissociated cell cultures of the ventral horn

To study the ROS amount and the expression of the DNA damage response proteins in motor neurons *ex vivo*, the ventral horn of the cervical spinal cord of Wobbler and wild-type mice from stable clinical phase (p40) was isolated and a dissociated motor neuronal cell culture was prepared according to Zwilling et al. (2020). Cells were seeded on glass slides and kept in culture for 10 days *in vitro* (div) with periodic medium changes every 48 h. For the entire time, the temperature was kept at 37°C and the CO_2_ level at 5%. The motor neurons to be studied were selected on the basis of their morphology (multipolar neurons) with a soma size of 60–90 μm and a nucleus size of about 20 μm as described earlier (Weber et al., 1997; Thomson et al., 2012). To investigate whether the application of ROS scavengers affects the amount of DNA damage response protein γH2ax in motor neurons, the following supplements were added to the cell culture medium:

(i) N-Acetyl-L-Cysteine (NAC; #sc202232, Santa Cruz, Dallas, Texas, USA) was dissolved in PBS at a concentration of 10 M and diluted to the final concentration of 20 μM directly in culture medium. Dissociated cells of the ventral horn were cultured for 8 days in normal culturing medium and for 48 h with supplemented medium.
(ii) Glutathione ethyl ester (#14953, Cayman Chemical, Ann Arbor, Michigan, USA) was dissolved in water at a concentration of 74.5 mM and diluted to the final concentration of 500 μM directly in culture medium. The dissociated cells of the ventral horn were cultured for 9 days in normal culturing medium and for 24 h with supplemented medium.
(iii) Mito-TEMPO (#sc-221945, Santa Cruz, Dallas, Texas, USA) was dissolved in DMSO at a concentration of 10 mM and diluted to the final concentration of 1 μM directly in culture medium. The dissociated cells of the ventral horn were cultured for 9 days in normal culturing medium and for 24 h with supplemented medium.

At least four animals per genotype were used for one procedure of motor neuron isolation. The entire experimental set-up was repeated at least five times per supplement.

### ROS measurement in dissociated motor neuronal cultures

For measurement of ROS amount in motor neurons *in vitro*, live-cell imaging was performed 10 days after cell isolation. The motor neurons to be studied were identified as described above (see Section “Motor neuron enriched dissociated cell cultures of the ventral horn”). After 15 min of live-cell imaging under normal conditions, medium was replaced by culturing medium including Dihydroethidium (DHE; final concentration: 10 μM) which reacts with superoxide anions resulting in a red immunofluorescence (Owusu-Ansah et al., 2008). The fluorescence signals of the cells were documented every 5 min for 1 h in DHE-including medium, 37°C and 5% CO_2_. Live-cell imaging was performed using a spinning disc confocal microscope (VisiScope Confocal-Cell Explorer, Visitron Systems GmbH, Puchheim, Germany) with a 20× objective (Plan-Apo 20×/0.75, Nikon Europe BV, Amsterdam, Netherlands). ImageJ 1.53f51 (National Institute of Health, USA) was used to analyze the average red fluorescence intensity within the soma of a motor neuron, while motor neurons themselves were detected in transmitted-light channel. A mean value of background fluorescence intensity without DHE addition was used for normalization. We prepared five different cell cultures per genotype for live cell imaging. Four different motor neurons of each motor neuronal preparation were subsequently documented over a period of 80 min.

### Immunofluorescence staining of DNA damage response proteins *in vitro*

To investigate the number of DNA damage response proteins in the nucleus of motor neurons, after 10 days in culture, the cells were fixed using 4% PFA for 15 min and washed with PBS afterwards. For permeabilization, glass slices with dissociated cells of the ventral horn were incubated with 0.3% Triton in PBS for 15 min at room temperature. After washing with PBS, unspecific binding sites were blocked with 2% goat serum in PBS for 30 min. The slices were then incubated with primary antibodies (rabbit-*α-*phospho-histone H2A.X (Ser139) antibody, #2577, Cell Signaling Technology, Danvers, Massachusetts, USA, 1:500 in 2% goat serum in PBS; chicken-*α-*NEFM Antibody, #PA1-16758, Thermo Fisher Scientific, Waltham, Massachusetts, USA, 1:1,000 in 10% goat serum in PBS) at 4°C over night. Thereafter, samples were reacted with secondary antibodies (α-rabbit-Alexa488, #11008, Thermo Fisher Scientific, Waltham, Massachusetts, USA, 1:300 in 2% goat serum in PBS; α-chicken-Alexa568, #A11041, Thermo Fisher Scientific, Waltham, Massachusetts, USA, 1:1,000 in 10% goat serum in PBS) for 2 h at room temperature. Nuclei were stained with DAPI (1 μg/ml; #D9542, Sigma-Aldrich, St. Louis, Missouri, USA) for 30 min at room temperature. Finally, the slices were coverslipped in fluoroshield (#F6937, Sigma-Aldrich, St. Louis, Missouri, USA) and stored at 4°C. Secondary antibodies were tested for specificity and showed no unspecific binding.

Samples were imaged using an inverted confocal Laser Scanning Microscope (LSM 800, Carl Zeiss Microscopy GmbH, Jena, Germany) equipped with the respective filter sets in combination with a 40× objective (Plan-Apochromat 40× /1.4 Oil, Carl Zeiss Microscopy GmbH, Jena, Germany). The motor neurons to be studied were selected as described above (see Section “Motor neuron enriched dissociated cell cultures of the ventral horn”). γH2ax in nuclei of the motor neurons was analyzed with aid of the microscopy image analysis software Imaris x64 (Version 9.3.1, Oxford Instruments, UK). The nuclear surface was segmented using the surface function. By means of the spots function (diameter over 0.2 μm and intensity quality above 19.2) immunosignals of the phospho-histone H2A.X signal were measured to analyze the amount of DNA damage response and thus double strand breaks in the nucleus of motor neurons (Dumur et al., 2019). At least 35 cell nuclei from at least five independent experiments were analyzed per genotype.

### GSH/GSSG assay

For measurement of the amount of GSH and GSSG in wild-type and Wobbler spinal cord (p40) the GSH/GSSG detection assay kit (#ab138881, Abcam, Cambridge, UK) was used according to manufacturer’s protocol. In brief, spinal cord tissue was lysed and homogenized in extraction buffer (#ab65349, Abcam, Cambridge, UK), centrifuged for 15 min at 4°C at 12,700 rpm and the supernatant was collected. For measurement of reduced glutathione (GSH) and total glutathione (GSH+GSSG), 50 μl of the samples were plated in duplicates in a 96-well plate suitable for fluorescence measurements. For GSH detection, 50 μl of GSH assay mixture were added to samples, for total glutathione (GSH+GSSG), 50 μl of total glutathione assay mixture were added to samples. After incubation at room temperature for 15 min protected from light, the fluorescence was measured at 490 nm excitation and 520 nm emission with a TriStar2 Multimode Reader LB 942 (Berthold Technologies GmbH and Co. KG, Bad Wildbad, Germany). Twelve animals per genotype were used.

### Statistical analysis

Statistical data analysis was performed with Prism 7.0 (GraphPad Inc., La Jolla, California, USA). Data represent mean values of at least three to seven independent experiments ± standard deviation (SD) or standard error of the mean (SEM). Kolmogorov-Smirnov normality test was used to confirm normal distribution. Data were tested for significance using Student’s t test or two-way ANOVA with a Sidak *post-hoc* test. Results with *p* < 0.05 were considered statistically significant.

## Results

### Increased expression of DNA damage response proteins—p53bp1 and γH2ax in spinal cord of symptomatic Wobbler mice

The initial question was whether an increased amount of ROS in the cervical part of the spinal cord of Wobbler mice at stable clinical stage observed in previous studies (Röderer et al., 2018) has a harmful effect on DNA by inducing the formation of toxic DNA DSBs. To get further insights into the cellular response to DNA DSB formation in cervical spinal cord of wild-type and Wobbler mice, we subsequently investigated the expression on protein- and mRNA-levels of two described participants in the response to DNA DSB formation—p53bp1 and γH2ax (Schultz et al., 2000).

**Figure 1:**
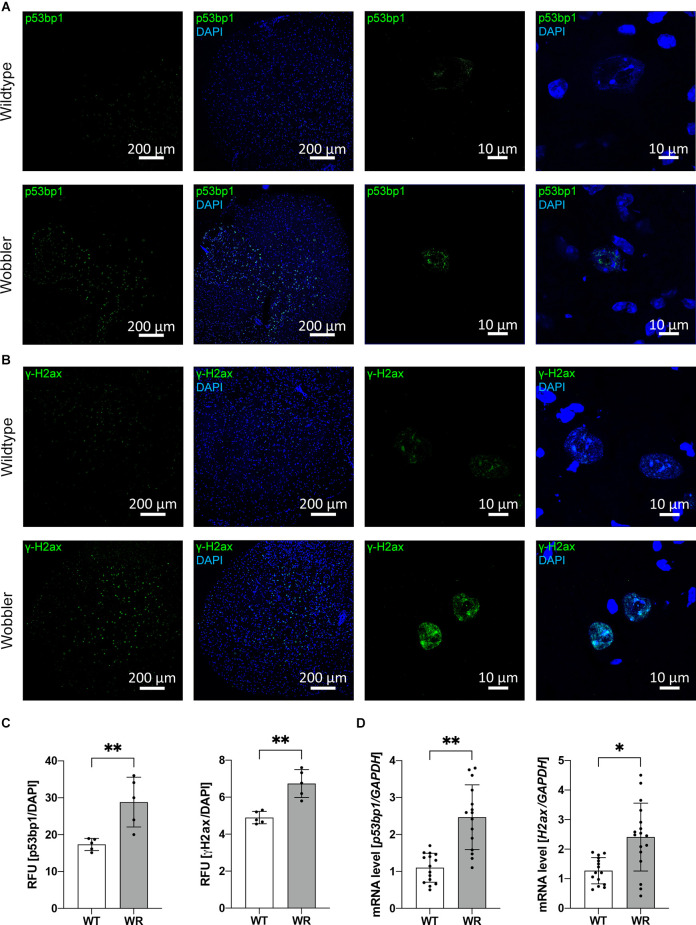
Increased expression of DNA damage response proteins, p53bp1 and γH2ax, in cervical Wobbler spinal cord. **(A,B)** Exemplary immunofluorescence staining of p53bp1 (green, **A**) and γH2ax (green, **B**) and DAPI (blue) in cryo-sections of cervical wild-type and Wobbler spinal cord (p40). **(C)** Relative fluorescence units of p53bp1 and γH2ax in cryo-sections of wild-type and Wobbler spinal cords (p40). ImageJ 1.53f51 (National Institute of Health, USA) was used to analyze the fluorescence intensity. Intensity of DAPI channel was used for normalization. Data are provided as means ± SD including individual data points. Data were tested for significance using Student’s t-test. Significant differences are indicated by ^**^p < 0.01, *n* = 5. **(D)** Tp53bp1- and H2ax-mRNA expression level in wild-type and Wobbler mice at p40. The qPCR was performed using five samples for each genotype. For relative quantification of p53bp1-, and H2ax-mRNA expression, the 2^−ΔΔCt^ method was conducted using the housekeeping gene GAPDH for normalization; data are provided as means ± SD including individual data points. Data were tested for significance using Student’s t-test. Significant differences are indicated by ^*^p < 0.05, ^**^p < 0.01, *n* = 5.

For investigation of the amount of p53bp1 and γH2ax protein, frozen sections were prepared, followed by immunofluorescence staining for the appropriate marker (Figures 1A,B). Thereby, a significantly higher relative signal intensity of both investigated DNA damage response proteins was observed in cervical spinal cord sections of Wobbler animals (Figure 1C). Noticeably, very large nuclei with a diameter of about 20 μm predominantly showed a fluorescent signal of p53bp1 or γH2ax staining (Figure 1A, enlarged images on the right). In addition, the mRNA expression of *Tp53bp1* and *H2ax* was analyzed by qPCR in cervical spinal cord samples. Concordantly, a significant upregulation of both mRNA level could be detected in Wobbler compared to wild-type spinal cord samples (Figure 1D).

### Elevated ROS levels and γH2ax spots in dissociated motor neurons of symptomatic Wobbler mice

In order to investigate the ROS levels in motor neurons *in vitro* without exogenous influences of the naturally occurring micromilieu, the spinal cord of 40-days old Wobbler and wild-type mice was isolated, and the cells were cultivated in dissociated cell culture for 10 days *in vitro* (div).

**Figure 2:**
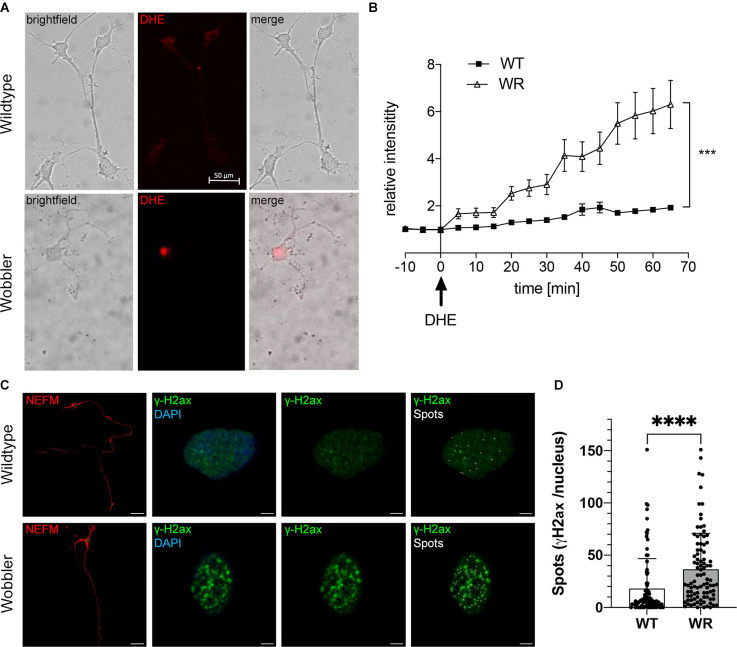
Elevated ROS levels in motor neurons of Wobbler mice correlate with increased abundance of the DNA damage response protein γH2ax. **(A)** Exemplary images of dissociated motor neurons from wild-type and Wobbler animals (p40+10div) 65 min after DHE (red fluorescence) addition and live cell imaging. Live-cell imaging was performed using a spinning disc confocal microscope with a 20× objective. The fluorescence signals of the cells were documented every 5 min for 1 h in DHE-including medium, 37°C and 5% CO_2_. Scale bar = 50 μm. **(B)** Relative red fluorescence intensity in five dissociated motor neuronal cell cultures from p40 wild-type and Wobbler mice documented in 5 min intervals of 20 cells per genotype. At timepoint 0 the normal medium was exchanged for DHE including medium. ImageJ 1.53f51 (National Institute of Health, USA) was used to analyze the average red fluorescence intensity within the soma of a motor neuron, while motor neurons themselves were detected in transmitted-light channel. A mean value of background fluorescence intensity without DHE addition was used for normalization. Data are provided as means ± SEM. Data were tested for significance using two-way ANOVA with a Sidak *post-hoc* test. Significant differences are indicated by ***p < 0.001, *n* = 20 cells from five dissociated cultures per genotype. **(C)** Exemplary images of wild-type and Wobbler motor neurons (p40+10div) stained with anti-NEFM antibody (red), anti-phospho-Histone H2A.X (green) and DAPI (blue). The overview images (1st row) show an exemplary motor neuron, with rows 2–4 focusing on the nucleus (blue) of the exemplary cell to see the H2ax signals (green) and spots (white) created using Imaris 9.3.1. Pictures were taken with a confocal fluorescent microscope equipped with a 40× oil objective. Scale bar (1st row) = 30 μm. Scale bar (2nd–4th raw) = 2 μm. **(D)** Detected spots of γH2ax staining in wild-type and Wobbler motor neuronal cell nuclei. Wobbler mice show a significantly higher number of spots with an average of 36.56 while wild-type motor neurons have an average number of spots of 18.09. Data are provided as means ± SD including individual data points. Data were tested for significance using Student’s t-test. Significant differences are indicated by ^****^p < 0.0001, *n* = 90–94 nuclei per genotype of a least five experiments.

To investigate intracellular ROS levels in isolated motor neurons, fluorescence intensity of DHE was measured in 20 motor neurons from four different cultures (p40+10 div) per genotype (Figure 2A). On average there was no autofluorescence in cell soma of wild-type and Wobbler motor neurons without DHE (Figure 2B, time period: −10 min to 0 min). After exchange to DHE-containing medium (Figure 2B, timepoint 0 min), the average intensity of red fluorescence in the cell soma of Wobbler motor neurons showed a significant progressive increase compared to the cell soma of wild-type motor neurons. After an hour of observation, the average fluorescence intensity in soma of wild-type motor neurons indicated an increase to 1.87 in relative comparison to the baseline, while Wobbler motor neurons showed a relative intensity of 6.3 (Figure 2B). Thus, Wobbler motor neurons were characterized by a strong and significant increase in DHE associated fluorescence 1 h after DHE addition *in vitro* compared to wild-type motor neurons. Thus, it can be concluded that motor neurons from Wobbler mice experience a significantly increased ROS level *in vitro* compared to wild-type.

To test whether elevated ROS levels in Wobbler motor neurons cause increased DNA damage, the protein γH2ax was investigated by immunofluorescent staining in dissociated motor neuronal cultures as a marker for the formation of DNA DSBs. With the aid of Imaris spots function the punctate signals of γH2ax were converted into spots (Figure 2C). Comparing γH2ax signals in wild-type and Wobbler motor neurons from 40-days old mice (p40+10 div), striking differences emerged. Nuclei of Wobbler motor neurons displayed a significantly higher number of spots than motor neurons of wild-type mice and thus more DSBs (Figures 2C,D). Based on these results, it can be hypothesized that the increased ROS levels observed in Wobbler animals give rise to the increased DNA damage and further to accumulation of γH2ax signal as a marker of DNA damage and repair.

### Dysregulated mediators of ROS detoxification mechanisms in Wobbler motor neurons

**Figure 3:**
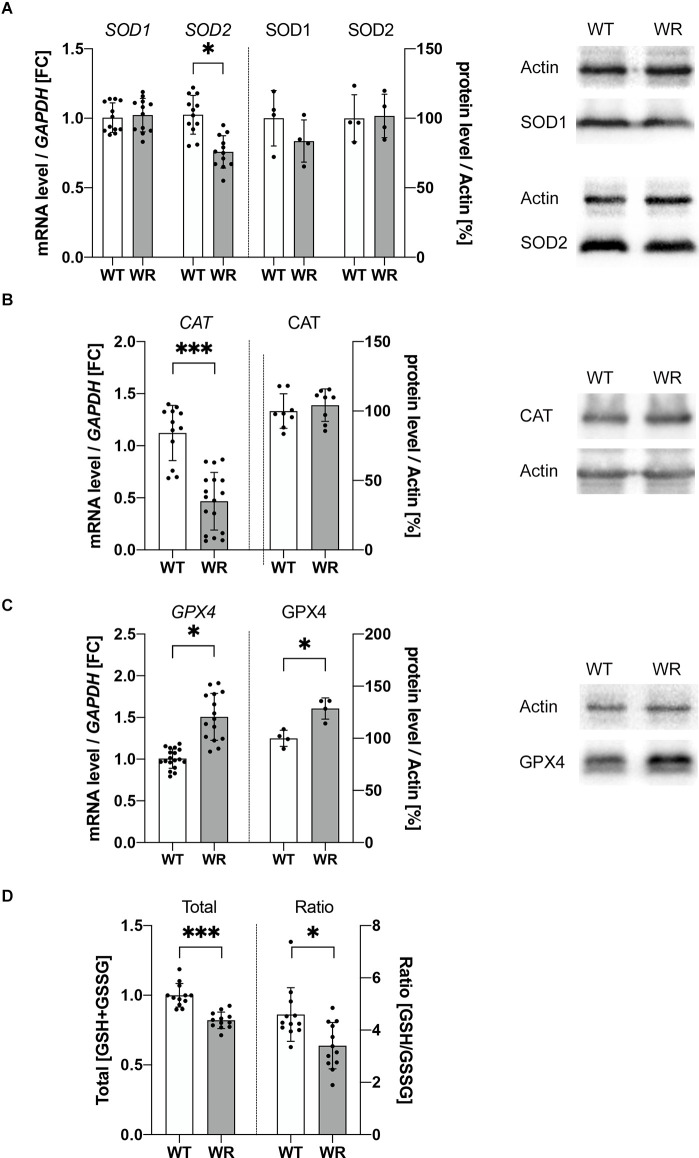
Dysregulated expression of ROS detoxifying molecules in cervical Wobbler spinal cord. **(A–C)** mRNA and protein expression levels of SOD1 and 2 **(A)**, CAT **(B)**, and GPX4 **(C)** at stable clinical stage (p40) in cervical spinal cord of wild-type (WT) and Wobbler (WR) mouse. For relative quantification of mRNA level, the 2^−ΔΔCt^ method was conducted using *GAPDH* for normalization. For semiquantitative analysis of protein expression levels, arithmetic analysis of the band intensity with ImageJ 1.53f51 (National Institute of Health, USA) software was used. Band intensities of proteins of interest were normalized to the housekeeper actin. Normalized protein levels were compared between different genotypes. All data are presented as the mean values ± SD including individual data points, and Student’s t-test was performed for significance testing between WT and WR. Significant differences are indicated by ^*^p < 0.05, ^***^p < 0.001, *n*_(qPCR)_ = 5; *n*_(WB)_ = 4–8. **(D)** The total amount of reduced and oxidized glutathione was measured in the spinal cord of 40-days old wild-type and Wobbler mice using a GSH/GSSG detection assay kit. Total [GSH+GSSG] was normalized to WT. Using the same GSH/GSSG detection assay kit a significantly decreased ratio of [GSH/GSSG] could be detected in Wobbler spinal cord compared to wild-type spinal cord. All data are presented as the mean values ± SD including individual data points, and Student’s t-test was performed for significance testing between different genotypes. Significant differences are indicated by ^*^p < 0.05, ^***^p < 0.001, *n* = 12.

As we detected increased DHE intensity in Wobbler motor neurons *in vitro*, indicating increased ROS levels, two hypotheses can be raised: (i) do motor neurons of Wobbler animals exhibit increased ROS production; or (ii) are there deficits in ROS detoxification? Therefore, further focus was placed on the analysis of important intracellular mediators of ROS detoxification mechanisms. For this purpose, mRNA and protein expression of several molecules for ROS detoxification were analyzed in cervical spinal cord of 40-days old wild-type and Wobbler mice (Figure 3). Evaluation of the expression of superoxide anion detoxifying proteins SOD1 and SOD2 showed no alteration in protein expression, although a significantly decreased mRNA level of SOD2 was detected in the cervical spinal cord of Wobbler mice (Figure 3A). Detoxification of hydrogen peroxide, the product of SOD, is carried out by catalase or the glutathione system. Here, the analysis of the mRNA expression of catalase revealed a strongly significant reduction of catalase, which, however, was not reflected at the protein level (Figure 3B). In contrast, the expression of GPX4, an important member of the glutathione system especially for motor neurons, was significantly increased at both the mRNA and protein level in cervical spinal cord of Wobbler mice (Figure 3C).

After becoming apparent that an increased expression of glutathione-consuming peroxidase was present in the spinal cord of Wobbler mice, investigations concerning the cofactor glutathione were performed. Therefore, the total amount and the ratio of oxidized (GSSG) and reduced glutathione (GSH) in Wobbler and wild-type cervical spinal cord was quantified. Wobbler spinal cord showed a significantly decreased amount of total glutathione (GSH+GSSG), in combination with a significantly decreased ratio of GSH/GSSG (Figure 3D) indicating deficits in ROS detoxification and oxidative stress in this tissue.

### Effects of ROS scavengers N-Acetyl-L-Cysteine (NAC), glutathione ethyl ester (GSHe), and Mito-TEMPO on Wobbler motor neurons *in vitro*

**Figure 4:**
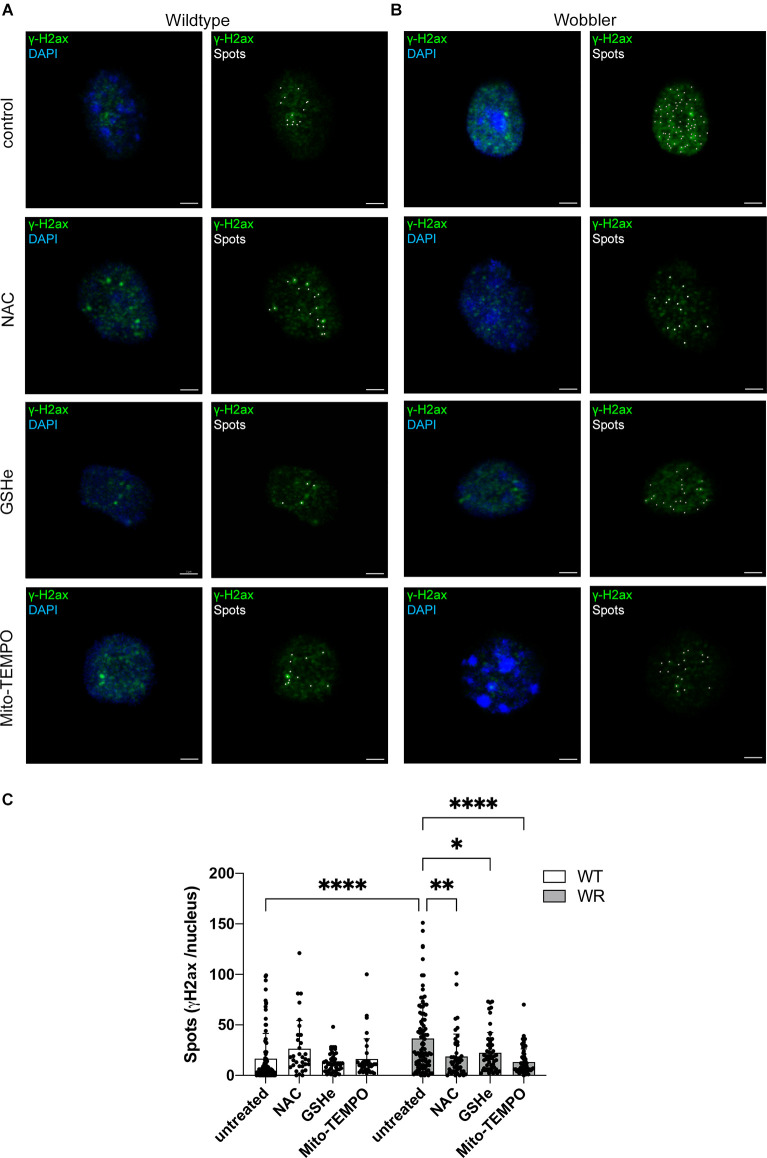
Altered γH2ax Spots in Wobbler motor neuronal nuclei after treatment with ROS scavengers NAC, GSHe, and Mito-TEMPO. **(A,B)** Exemplary images of untreated control and NAC-, GSHe-, and Mito-TEMPO-treated wild-type **(A)** and Wobbler **(B)** motor neurons. Nuclei were stained with DAPI (blue) and DNA damage response protein γH2ax (green). Pictures were taken with a confocal fluorescent microscope equipped with a 40× oil objective. Amount of γH2ax and thus double strand breaks was analyzed with the spots function (white) of Imaris 9.3.1. Scale bar = 2 μm. **(C)** Evaluation of the spots number of untreated control and NAC-, GSHe- and Mito-TEMPO-treated wild-type (WT) and Wobbler (WR) motor neurons at stable clinical phase (p40+10div). Data are provided as means ± SD including individual data points. Data were tested for significance using two-way ANOVA with a Sidak *post-hoc* test. Significant differences are indicated by ^*^p < 0.05, ^**^p < 0.01, ^****^p < 0.0001, *n* = 35–55 nuclei per genotype of a least five experiments.

After observing a significant difference in the expression of several key ROS detoxification representatives including an altered ratio of the redox partners GSH/GSSG in the Wobbler spinal cord, dissociated motor neurons of 40-days old wild-type and Wobbler mice were cultured and treated with the ROS scavengers N-acetyl-L-cysteine (NAC), glutathione ethyl ester (GSHe), and Mito-TEMPO after a defined time period (see above). Here, immunofluorescence staining for γH2ax as a DNA double-strand-break-repair-related protein was analyzed and the intensity of the signals was evaluated using the spot function of Imaris 9.3.1 (Figures 4A,B).

Considering the evaluated spots in motor neurons from wild-type animals, no significant difference in the number of spots after treatment with any of applied ROS scavenger was observed within this genotype (Figure 4C). In contrast, looking at the influence of ROS scavenger treatment within Wobbler motor neurons, it was obvious that all tested ROS scavengers displayed a significant decrease in the number of detected γH2ax spots (Figure 4C).

## Discussion

ALS is a highly heterogenous disease considering the underlying pathology. To date, no significant therapeutic option exists that could stop the progressive loss of motor neurons over a prolonged period of time (Brown and Al-Chalabi, 2017). However, it is known that the majority of all ALS patients exhibit uniform phenotypes reflected in signs of oxidative stress (Tam et al., 2019), making it an important factor in the pathogenesis of ALS (Matschke et al., 2019; Singh et al., 2019). Protein and lipid peroxidation as well as oxidative DNA damage could be observed in the spinal cord of ALS patients (Ferrante et al., 1997; Shibata et al., 2001). Previous studies also indicate changes in the ROS homeostasis in an animal model for ALS, the Wobbler mouse (Moser et al., 2013; Röderer et al., 2018). Thus, own previous work identified increased ROS level in the cervical spinal cord of the Wobbler mouse (Röderer et al., 2018). A neuroprotective and symptom-improving effect of several ROS scavengers on the Wobbler mouse has already been described (Henderson et al., 1996; Abe et al., 1997; Ikeda et al., 2015; Zwilling et al., 2020). However, an accurate analysis of the ROS homeostasis, especially in Wobbler mouse motor neurons, was still lacking. The present study was conceptualized to further substantiate and concretize existing findings and to be able to assess the consequences of an altered ROS homeostasis for single motor neurons.

The DNA of the cell is highly susceptible to changes in the intracellular ROS level, and it is essential for cell survival to detect and effectively repair oxidative damage (Ba and Boldogh, 2018). Base oxidation, DNA single-strand-breaks (SSBs) and consecutive DNA DSBs are a result of increased exposure of DNA to elevated ROS concentrations with a consequence of increased overall DNA instability (Kobayashi et al., 2017; Ba and Boldogh, 2018; Qi et al., 2019). Two central biomarkers for detecting DNA DSBs have been described in the past: γH2ax and p53bp1 (Schultz et al., 2000; Mah et al., 2010; Kannan et al., 2018; Williamson et al., 2020). In the presence of DNA damage one of the earliest key factors of the DNA repair Histone H2ax is phosphorylated to γH2ax thereby allowing the assembly of DNA repair-related proteins to DNA (Kobayashi, 2004; Kuo and Yang, 2008; Podhorecka et al., 2010). Moreover, hyperphosphorylated p53bp1 attaches to the newly formed DNA DSB and initiates non-homologous end joining (NHEJ) *via* the recruitment of repair factors, which play a major role especially in non-dividing cells such as neurons (Iyama and Wilson, 2013; Panier and Boulton, 2014).

On mRNA level, we could observe a significantly increased expression of both p53bp1 and unphosphorylated H2ax in the cervical spinal cord of the Wobbler mouse from stabilized clinical phase (p40). This observation is verified by evaluation of protein expression of p53bp1 and γH2ax in the cervical spinal cord, where an increased signal of p53bp1 or γH2ax was detected mainly in very large nuclei with a diameter of about 20 μm of the spinal cord. Although a statement on the colocalization of p53bp1 and γH2ax is not provided by our experimental setup, signals of both investigated proteins are detectable in cells with the same morphology, which can be identified as motor neurons due to their large nucleus. Although elevated phosphorylation of H2ax does not necessarily correlate to increased mRNA expression of H2ax, this result may indicate a functional work flow of DNA DSB detection and accumulation (Zhang et al., 2016). All in all, the increased mRNA expression of both p53bp1 and unphosphorylated H2ax in the spinal cord as well as the increased protein level in cervical spinal cord of Wobbler mice strongly suggest that there is an increased accumulation of DNA DSBs in Wobbler tissue presumably due to oxidation of DNA.

To investigate these results more extensively in motor neuronal cells, dissociated motor neuronal cell cultures were performed. Due to the isolated cultivation of the cells *in vitro*, the motor neurons do not experience any influence from neighboring cells, like microglia, and thus the endogenous factors of cell homeostasis can be observed (Zwilling et al., 2020). Wobbler motor neurons from stabilized clinical phase (p40+10div) were incubated with dihydroethidium (DHE). DHE diffuses well through cell membranes and reacts most strongly with superoxide anions but weaker also with other ROS species and NO resulting in a red immunofluorescence and thus is a marker for oxidative stress (Owusu-Ansah et al., 2008; Gardiner et al., 2020). Wobbler motor neurons show an almost three times higher increase in fluorescence intensity after 65 min *in vitro* compared to wild-type motor neurons. This suggests that the increased ROS levels measured in the Wobbler spinal cord by Röderer et al. (2018) is also found in isolated motor neurons.

As a consequence of increased ROS levels, we next examined the occurrence of DNA damage in isolated motor neuronal cultures. Therefore, we measured the immunofluorescence signals of γH2ax in the nucleus of dissociated motor neurons of Wobbler and wild-type mice. Wobbler motor neurons in dissociated cell culture displayed a significantly higher number of γH2ax foci compared to wild-type. This result is interesting in two aspects: first, it provides further evidence of increased DNA DSB abundance in motor neurons of Wobbler mice and confirms the overexpression of *H2ax* at the mRNA level and increased γH2ax fluorescence signal intensity in spinal cord sections of Wobbler mice. Second, our observation provides evidence for the influence of endogenous factors, like ROS formation, in the emergence of toxic DNA DSBs in motor neurons. Here, a link can be drawn to the increased ROS levels in dissociated motor neuronal cells of Wobbler mice. These results may be indicative of increased oxidative stress in Wobbler spinal cord, particularly in motor neurons, and its dependence on endogenous factors.

The combination of increased expression of both p53bp1 and *H2ax*/γH2ax generally indicates an increased number of DNA DSBs (Kannan et al., 2018). In this study, we investigated p40 mice, as previous studies from our group have shown increased ROS levels in the spinal cord only at this stage. Based on the unchanged ROS level of p0 and p20 Wobbler animals (Röderer et al., 2018), we assume that ROS-related DNA damage is not yet detectible at the time points of the development earlier than p40. However, further investigations on the time-resolved development of the Wobbler phenotype are necessary to clarify the relation between ROS induction and DNA damage in ALS.

Various studies have already shown an increased activation of DNA damage response in human ALS and suggest that the accumulation of DNA damage response markers may play a role in premature cell death (Lopez-Gonzalez et al., 2016; Farg et al., 2017; Kim et al., 2020). Among others, the analysis of Kim et al. (2020) in *postmortem* tissue samples of ALS patients and induced pluripotent stem cells interestingly showed an accumulation of DNA damage like single-stranded DNA and 8-oxoguanin as well as an increased recruitment of the DNA damage and repair components like ATM Serine/Threonine Kinase (ATM) and Breast Cancer Anti-Estrogen Resistance 1 Cas Family Scaffold Protein (BCRC1). Similar observations of increased numbers of DNA damage, like DNA double strand breaks, were shown in different ALS subgroups. Thus, an increased number of p53bp1 and γH2ax expression was shown to be associated with specific *C9Orf72* variants (Farg et al., 2017). This increased number of DNA damage response proteins was also associated with increased oxidative stress in an iPSC model with *C9Orf72* variants (Lopez-Gonzalez et al., 2016). Konopka et al. (2020) demonstrated that TDP-43 ALS-associated mutations lead to a reduced activity of NHEJ, which in consequence leads to increased DNA damage. Therefore, it is important to emphasize that despite different genetic backgrounds, similar effects on genomic DNA have been described in the different ALS models. Histone 2ax has also been shown to play a key role, in both, the registration of increased ROS levels and in the initiation of various DNA damage response components (Kuo and Yang, 2008; Podhorecka et al., 2010; Barral et al., 2014; Merighi et al., 2021). Interestingly, H2ax-knock out mice exhibit dysfunctional motor characteristics, such as an impaired overall motor activity as well as impaired motor balance, among others (Weyemi et al., 2018). Thus, the increased expression and phosphorylation of *H2AX*/γH2ax in Wobbler mice observed in this study indicates a response to existing oxidative stress to trigger a mechanism that, as a consequence, should counteract the loss of function of motor neurons.

Maintaining ROS-homeostasis is essential to maintain normal cell function. Therefore, the cell is equipped with various antioxidant mechanisms to scavenge ROS (Lagouge and Larsson, 2013; Pourahmad et al., 2016; Matschke et al., 2019). Physiologically occurring superoxide radicals are converted to hydrogen peroxide by two intracellular superoxide dismutases (SODs), cytosolic Cu-Zn SOD (SOD1) and mitochondrial MnSOD (SOD2; van Remmen et al., 2003; Lagouge and Larsson, 2013). Another important factor for neuroprotection is the detoxification of hydrogen peroxide produced by SODs (Ren et al., 2017; Matschke et al., 2019). The highly reactive hydrogen peroxide is detoxified by catalases and the glutathione system (Ren et al., 2017; Matschke et al., 2019).

The expression and activity level of SODs plays an important role in balancing the concentration of ROS (Miao and St Clair, 2009). With regard to human ALS, a primary mutation in the SOD1 gene can be found in nearly 15% of all European fALS and about 1% of European sALS cases (Zou et al., 2017; Mejzini et al., 2019). Interestingly, our results show no difference in the SOD1 expression between Wobbler and wild-type spinal cord. Although *SOD2* mRNA is significantly downregulated in Wobbler animals, there is no effect on protein expression in symptomatic stage Wobbler tissue. SOD2 plays a role in the mitochondrial antioxidant system and a reduction in SOD2 activity has already been associated with increased oxidative levels and cancer potential (van Remmen et al., 2003; Celotto et al., 2012). The question arises whether protein expression of SOD2 could also be found decreased at a later stage of age leading to mitochondrial oxidative stress and consequently to motor neuronal death. However, as we detect decreased *SOD2* mRNA and no deregulation of the protein level at a stage when all symptoms of ALS are already developed in the Wobbler animals and motoneuronal dysfunctionalities are already apparent, an underlying influence of a possible under-expression of the SOD2 protein at later age stages can be excluded.

Our results demonstrated a strong downregulation of *CAT* mRNA expression in cervical Wobbler spinal cord again without affecting protein expression. It should be emphasized that catalase activity is enhanced by ATM and p53, among others (Glorieux et al., 2015). Based on our results regarding the increased amount of γH2ax and p53bp1 in Wobbler, it is highly likely that catalase could receive a strong induction of its activity by activated ATM and p53.

Another important component in the detoxification of hydrogen peroxide are glutathione peroxidases, which consist of a group of seven isoforms (GPX 1–7). Of particular interest for our study was the increased expression of the ubiquitous GPX4 isoform, as it has been described to be strongly neuroprotective *in vitro* and *in vivo* (Seiler et al., 2008). Moreover, GPX4 is the only glutathione peroxidase that accepts protein thiol groups as reducing substrates under GSH deficiency (Seiler et al., 2008). Here, we show a significantly increased GPX4 expression at both, the mRNA and protein level in the spinal cord of p40 Wobbler mice. This may be an attempt of the cell to compensate for the reduced amount of GSH and reduced GSH/GSSG ratio that we have demonstrated, since GPX4 is the only isoform that does not rely on GSH as a reactant (Seiler et al., 2008).

Considering the general increase in ROS levels throughout the spinal cord and the increased ROS levels in isolated motor neurons, it is possible to hypothesize that the mechanisms of ROS-detoxification are not effective enough to detoxify ROS and thus ensure neuroprotection. It is striking here that catalase, in contrast to glutathione peroxidases, is not increased in expression at the protein level, although it receives an expression stimulus *via* increased ROS concentration. The non-increased expression of catalase is even more remarkable because it has been shown in the past that 50% of hydrogen peroxides are detoxified by catalase, whereas the other half are detoxified by glutathione peroxidases (Sepasi Tehrani and Moosavi-Movahedi, 2018). Catalases in Wobbler mice should receive an additional expression stimulus from p53. Thus, increased expression of p53 has been described in previous publications in the Wobbler mouse and is considered an important inducer of catalase expression (Eve et al., 2007; Kang et al., 2013).

Furthermore, we also analyzed the relative and absolute concentrations of oxidized and reduced glutathione, which is an important redox partner for glutathione peroxidase in particular. Glutathione (GSH), a necessary cofactor for GPX function, is synthetized by the cytosolic y-glutamylcysteine ligase and GSH synthetase in virtually all cell types with the liver as the major producer and exporter (Wu et al., 2004; Chi et al., 2007; Aoyama and Nakaki, 2013). Neurons do not have an uptake mechanism for GSH and rely on the uptake of cysteine and thus their own synthesis of GSH to maintain adequate concentrations (Johnson et al., 2012; Aoyama and Nakaki, 2013; Rae and Williams, 2017). GSH is involved in maintaining reducing conditions in all cells as an important antioxidant (Townsend et al., 2003). In case of reduction, the thiol-group of the cysteine is oxidized, and two glutathione molecules react to glutathione disulfide (GSSG). GSH/GSSG is the major redox couple in neurons (Wu et al., 2004; Ghosh et al., 2014). In p40 Wobbler spinal cord we measured a significantly decreased level of total glutathione [GSH+GSSG] as well as a significantly decreased ratio [GSH/GSSG]. This reconfirms the underlying oxidative stress in cervical Wobbler spinal cord, with a reduced amount of total glutathione and a shifted ratio to the oxidized form. This reduced glutathione level also suggests that there is not enough GSH available as a coenzyme for proper glutathione peroxidase function. On the one hand, GPX4 could be reflectively upregulated to compensate the lacking glutathione, and on the other hand the assumption arises that lacking reduced glutathione nevertheless leads to a reduced efficiency of glutathione peroxidase and thus to an increased ROS concentration.

Based on the abnormalities we observed in the GSH-dependent ROS detoxification system, we further specified these results using glutathione-based ROS scavengers. Therefore, we treated dissociated motor neurons of wild-type and Wobbler spinal cord (p40+10div) with N-acetyl-L-cysteine and glutathione ethyl ester (GSHe) for a defined period of time and measured the number of γH2ax spots and thus the DNA damage response afterwards.

N-acetyl-L-cysteine (NAC) is a well-described antioxidant. It has direct antioxidant effects *via* the modulation of the transcription factor NF-KB (Staal et al., 1995; Oka et al., 2000) and indirect antioxidant effects as a precursor of cysteine and thus GSH (Henderson et al., 1996; Aldini et al., 2018). After NAC is transported into the cells, it is deacetylated to cysteine (Aldini et al., 2018). As neurons rely on the uptake of cysteine for GSH production and thus, cysteine is the rate-limiting factor in the glutathione synthesis, NAC can replenish the GSH content (Rushworth and Megson, 2014). In a prior study the degeneration of Wobbler mice motor neurons could significantly be decreased by treating the mice directly with NAC (Henderson et al., 1996). Furthermore, a ROS-reducing effect of NAC in primary neuronal cells has already been shown (Wu et al., 2018). In this study, we observed a significantly decreased number of γH2ax spots and thus DNA damage in Wobbler motor neurons after treatment with NAC. Assuming an increased ROS-level and a decreased level of glutathione, especially the reduced form, as an important antioxidant in Wobbler motor neurons, it can be concluded that NAC, as a precursor of glutathione, can replenish the GSH content and thus reduce oxidative stress in Wobbler motor neurons resulting in less oxidative DNA damage. Furthermore, direct effects of NAC, through its influence on gene modulation, could also lead to a decrease in γH2ax spots.

Treating motor neurons with GSHe also lead to a reduced number of γH2ax spots and thus, DNA damage in Wobbler motor neurons (p40+10div). GSHe is a ROS scavenger which increases intracellular GSH and bypasses the need of GSH synthesis. GSHe is cell-permeable and readily de-esterified by intracellular esterases (Anderson et al., 1985; Dringen et al., 1999; Chen et al., 2018). In prior studies, a neuroprotective effect of GSHe-treatment on ischemic damaged brain and spinal cord injuries in rats has already been shown (Anderson et al., 2004; Guízar-Sahagún et al., 2005). This neuroprotective effect is associated with a reduction in ROS levels (Ross et al., 2012; Rush et al., 2012). GSHe treatment of ALS mouse models expressing the ALS-associated SOD1 mutation delayed early degenerative changes in motor neurons and reduced oxidative stress (Winkler et al., 2014).

Thus, our results confirm the hypothesis that increased ROS levels in motor neurons of Wobbler animals are associated with impaired ROS detoxification by the glutathione system.

Finally, it remained to consider whether motor neurons from Wobbler animals also show increased ROS production. As mitochondria are physiologically the major producers of intracellular ROS, mitochondrial dysfunction can lead to many cell damaging processes including an excessive production of this harmful species (Smith et al., 2019). Since we demonstrated in a previous study that mitochondria in Wobbler motor neurons have abnormal morphology (Stein et al., 2021) and mitochondrial malfunction is known to be present in Wobbler animals (Xu et al., 2001; Dave et al., 2003; Santoro et al., 2004; Gonzalez Deniselle et al., 2012), it is tempting to assume an increased mitochondrial ROS production in a Wobbler phenotype. Therefore, an additional ROS scavenger, Mito-TEMPO, was used to specifically scavenge mitochondrially produced ROS. Mito-TEMPO consists of the antioxidant piperidine nitroxide (TEMPO) and the lipophilic cation triphenylphosphonium (TPP+). Due to the combination with TPP as a membrane-permeant cation that is accumulated several hundred-fold within mitochondria driven by the membrane potential the antioxidant TEMPO as a SOD mimetic can accumulate within mitochondria (Trnka et al., 2008). Therefore, it helps protect against oxidative damage to the mitochondria and has superoxide and alkyl radical scavenging properties (Trnka et al., 2008; Du et al., 2017). Mito-TEMPO has already been shown to have protective effects on neuronal cells in the neurodegenerative disease Alzheimer’s disease, in which oxidative stress also plays an important role (Lu et al., 2015; Hu and Li, 2016). When primary cortical neurons were treated in cell culture with Mito-TEMPO, a reduction in ROS concentration could also be observed (Zhou et al., 2015). More specifically, in primary cultured neurons, Aß-promoted mitochondrial superoxide production and neuronal lipid peroxidation were significantly suppressed by Mito-TEMPO (Hu and Li, 2016). In this study, we demonstrated that treating Wobbler motor neurons with Mito-TEMPO also leads to a significantly decreased number of γH2ax foci and thus DNA damage in motor neurons. Hence, counteracting mitochondrial ROS production seems to be an important part in reducing DNA damage in Wobbler spinal cord. In conclusion, not only glutathione-based but also mitochondria-targeted ROS scavengers might be involved in reducing oxidative stress and thus the extent of DNA damage in the Wobbler spinal cord.

Using ROS scavengers, we were able to detect a reduced number of DNA damage response proteins in the nuclei of dissociated motor neurons from Wobbler mice. Thus, we hypothesize that scavenging mitochondria-produced ROS or enhancing the GSH system with GSH precursors or derivatives may reduce oxidative stress in motor neuron cells. As a result, the cellular ROS level and thus oxidation of DNA bases are reduced. This would lead to decreased number of SSBs and reduce the probability for an occurrence of DSBs resulting from the accumulation of SSBs. Furthermore, DSBs were described in the cell to trigger apoptosis (Ghardi et al., 2012). Since several studies revealed a positive effect of ROS scavengers against apoptotic cell death, it is probable to hypothesize that ROS scavengers not only reduce the number of toxic DSBs in the post-mitotic cell, but also reduce apoptotic cell death. However, this correlation still needs to be investigated in more detail in the ALS model of Wobbler mouse.

## Conclusion

ALS is the most common motor neuronal disease, which has been challenging both basic and clinical research and clinical therapy for decades. In this study, we present for the first-time evidence for increased DNA damage of symptomatic Wobbler mice, which might be a consequence of exposure to oxidative stress. In dissociated motor neuronal cultures, Wobbler motor neurons exhibit not only increased ROS levels, but also increased numbers of γH2ax foci and thus DNA DSBs, which could be counteracted by using glutathione precursors, derivatives, and a mitochondria-specific ROS scavenger Mito-TEMPO. The beneficial effect of Mito-TEMPO observed in our study also emphasizes the role of mitochondria in the pathology of increased ROS levels. In addition, a decreased amount of total glutathione and a decreased ratio of GSH/GSSG were detected in Wobbler spinal cord tissue, verifying the presence of oxidative stress presumably due to a fundamental glutathione deficiency.

Overall, this work reveals several pathologies in the Wobbler spinal cord and especially motor neurons, all of which are closely related to and might cause progressive, premature cell death. This substantiates previous work on the Wobbler mouse, which only described the benefit of antioxidant molecules *in vivo* without showing a possible link to oxidative damage to cellular components. Our work here focuses on the damage of DNA by oxidative stress in motoneurons and at the same time presents that it is a reversible process.

Our results indicate that a focused intervention in one pathomechanism might be insufficient in ALS therapy, as multiple systems are affected, so that multimodal therapy will be necessary to prolong the average lifespan of motor neurons and thus slow down the progression of the disease.

## Data Availability Statement

The original contributions presented in the study are included in the article/Supplementary material, further inquiries can be directed to the corresponding author.

## Ethics Statement

The animal study was reviewed and approved by Landesamt für Natur, Umwelt und Verbraucherschutz Nordrhein-Westfalen (LANUV).

## Author Contributions

VM designed, conceptualized, and supervised the research. MJ, FJ, HC, DS, and VM performed all experiments with support of VB and KW (IF visualization and analysis). MJ, FJ, HC, and VM analyzed, validated, and visualized the results. CT and JM contributed expertise (IF) and intellectual feedback. MJ, FJ, and VM wrote the original manuscript draft. CT and JM critically revised and edited the manuscript. All authors contributed to the article and approved the submitted version.

## Funding

KW is supported by the German Research Foundation (FOR 2848 and Germany’s Excellence Strategy—EXC 2033—390677874—RESOLV). This study was supported by German Academic Exchange Service and International Graduate School of Neuroscience (IGSN), Ruhr University Bochum to HC. JM is supported by the Federal Ministry of Education and Research (BMBF, 02NUK061B).
