# Supplementary material for: ROS scavengers decrease γH2ax spots in motor neuronal nuclei of ALS model mice in vitro
Source: Front Cell Neurosci. 2022 Aug 31;16:963169. doi: 10.3389/fncel.2022.963169 (PMC9470831; doi:10.3389/fncel.2022.963169)
Supplement: Supplementary file 1 [file Data_Sheet_1.PDF]

Supplementary Material

Actin ~ 42 kDa - #A-5060, Sigma-Aldrich

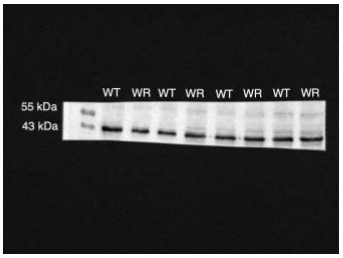

SOD1 ~ 23 kDa – sc-101523, Santa-Cruz

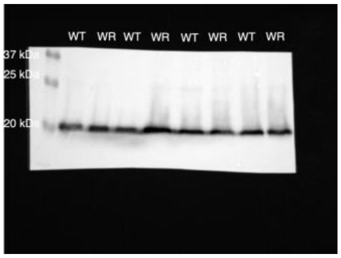

Actin ~ 42 kDa - #A-5060, Sigma-Aldrich

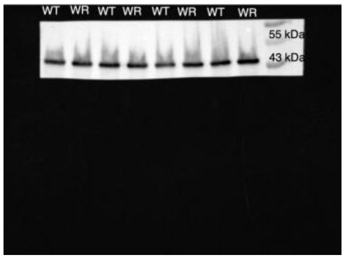

SOD2 ~ 25 kDa – sc-13314, Santa-Cruz

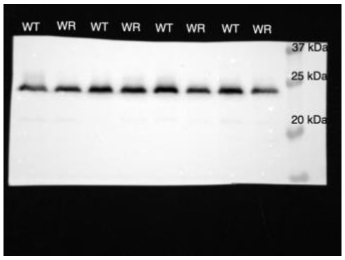

Actin ~ 42 kDa - #A-5060, Sigma-Aldrich

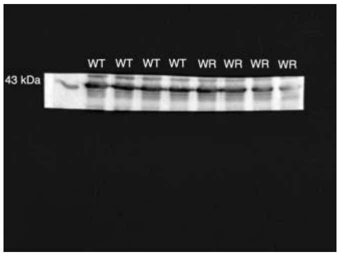

CAT ~ 64 kDa – sc-271803, Santa-Cruz

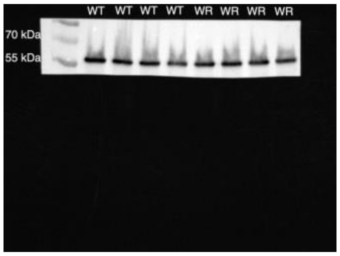

Actin ~ 42 kDa - #A-5060, Sigma-Aldrich

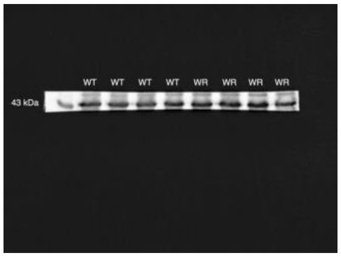

CAT ~ 64 kDa – sc-271803, Santa-Cruz

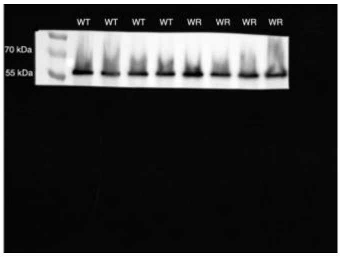

Actin ~ 42 kDa - #A-5060, Sigma-Aldrich

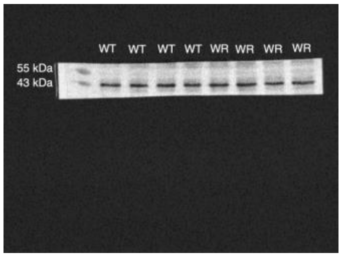

GPX4 ~ 21 kDa – sc-166570, Santa-Cruz

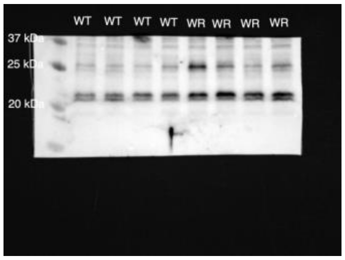

Supplementary Figure 1. All performed and analyzed full-length blots for figure 3.
